# Mapping the use of extended reality (XR) in radiation oncology education: a scoping review protocol

**DOI:** 10.1136/bmjopen-2024-094791

**Published:** 2025-06-16

**Authors:** Ching-Hsin Lee, Po-Jui Chen, Hung-Yi Lai, Mi-Mi Chen, Sze-Yuen Yau, Ching-Yi Lee

**Affiliations:** 1 Department of Radiation Oncology, Proton and radiation therapy center, Chang Gung Memorial Hospital at Linkou, Taoyuan, Taiwan; 2 Department of Neurosurgery, Chang Gung Memorial Hospital at Linkou, Taoyuan, Taiwan; 3 College of Medicine, Chang Gung University, Taoyuan, Taiwan; 4 Chang Gung Medical Education Research Centre, Taoyuan, Taiwan

**Keywords:** RADIOLOGY & IMAGING, MEDICAL EDUCATION & TRAINING, Virtual Reality, Augmented Reality

## Abstract

**Abstract:**

**Introduction:**

As the field of radiation oncology continues to evolve with rapidly advancing technologies, the need for innovative educational methods is critical. Extended reality (XR) technologies—including virtual reality, augmented reality and mixed reality—have emerged as transformative tools in medical education. While the potential of XR in healthcare education is recognised, there is a lack of comprehensive exploration specifically in the context of radiation oncology education. This scoping review aims to map the existing literature on XR technologies in radiation oncology training and education, identify barriers to their adoption and highlight opportunities for broader integration into curricula.

**Methods:**

This scoping review will follow the Arksey and O'Malley framework with enhancements by Levac *et al* and will adhere to the Preferred Reporting Items for Systematic Reviews and Meta-Analyses extension for Scoping Reviews guidelines. A comprehensive search will be conducted across databases, including MEDLINE, Scopus and Web of Science, to identify relevant studies on the use of XR technologies in radiation oncology education. Studies will be selected based on predetermined inclusion criteria using the population, concept, context framework. Data extraction will focus on the types of XR technologies used, educational settings, learning outcomes, barriers to adoption and methodologies for evaluating XR effectiveness. The results will be synthesised through descriptive statistics and qualitative thematic analysis. A consultation phase will engage experts to refine findings and ensure the practical relevance of the review.

**Ethics and dissemination:**

This protocol does not require ethics approval at the current stage as it involves a scoping review of publicly available literature. Ethics approval will be obtained prior to initiating the consultation phase involving experts. Written informed consent will be obtained from all individual participants included in the study. The study will be conducted in accordance with relevant guidelines and regulations and was approved by the Chang Gung Medical Foundation Institutional Review Board on 13 May 2025 (ref.: 202500731B0). The findings of this review will be disseminated through peer-reviewed publications, conference presentations and tailored executive summaries aimed at educators, policymakers and stakeholders in radiation oncology education.

**STRENGTHS AND LIMITATIONS OF THIS STUDY**

- This scoping review follows the Preferred Reporting Items for Systematic Reviews and Meta-Analyses extension for Scoping Reviews and Arksey and O’Malley framework with Levac enhancements.
- A comprehensive search strategy across multiple databases and grey literature sources will be employed.
- Data extraction and synthesis will be guided by a piloted standardised form and inductive thematic analysis.
- Inclusion of a consultation exercise with domain experts will enhance the practical relevance of findings.
- This review excludes non-English studies, which may limit the global generalisability of results.

## Introduction

Radiation oncology is a highly specialised medical field that integrates both the science of radiation and its therapeutic application in cancer treatment.^1^,^3^ Education and training within this discipline require a delicate balance between theoretical knowledge, practical skills and the use of advanced technologies, ensuring that practitioners are competent in both the technical aspects of treatment delivery and the holistic care of patients.^1 2^ The field of radiation oncology has experienced rapid advancements, including the development of advanced imaging techniques, precision therapy and personalised treatment plans.^3^,^5^ Continuous education and training are vital to keep healthcare professionals up to date with these advancements.^2 3 6 7^ With the growing complexity of cancer care and the rapid advancement in radiation therapy technologies, the need for innovative educational methods in this field has never been more pressing.^8^,^10^ Traditionally, radiation oncology education relied heavily on traditional lectures and hands-on clinical training.^11^ Modern radiation oncology education incorporates various educational methodologies designed to enhance learning outcomes and clinical competencies. Simulation-based training, for instance, has gained prominence as an effective educational tool.^3 6 12^ It allows learners to practise clinical scenarios in a controlled, risk-free environment, improving their technical skills and clinical decision-making abilities. E-learning platforms have also become integral to radiation oncology education, providing flexible and accessible learning opportunities.^12 13^

Extended reality (XR), a term encompassing virtual reality (VR), augmented reality (AR) and mixed reality (MR), is emerging as a promising tool to enhance learning and training in radiation oncology.^14 15^ XR is transforming medical education across many disciplines, offering immersive, interactive and safe environments for learners to acquire and practice skills.^16 17^ In radiation oncology, XR technologies could play a pivotal role in simulating complex treatment planning, dose calculations and patient positioning, which are critical components of radiation therapy.^14 15^ Additionally, XR allows learners to engage in virtual treatment sessions, where they can observe and perform procedures without the risk of patient harm.^18 19^ This technology has the potential to bridge the gap between traditional didactic teaching and hands-on clinical experience, providing a valuable supplement to existing educational methods.^14 18^

The use of XR in medical education is part of a broader trend of incorporating digital technologies to enhance learning.^17^ In fields such as surgery, XR has been used to simulate operative procedures, providing trainees with the opportunity to practice skills in a risk-free environment.^20^ A scoping review by Zhang *et al* on XR-assisted surgery highlighted how virtual environments are now routinely used for preoperative planning, intraoperative navigation and postoperative recovery.^21^ In a similar vein, the potential for XR in radiation oncology lies in its ability to simulate treatment delivery, visualise radiation beams and enhance the understanding of spatial relationships within the human body.^22^ A scoping review by Marvaso *et al* explores the applications of VR and AR in radiotherapy, specifically focusing on their potential to enhance educational experiences for professionals and patients.^14^ The review highlights how these technologies are being used to simulate radiotherapy procedures, such as external beam radiation therapy and brachytherapy, providing a risk-free environment for learners.^14^ While the study acknowledges the benefits of VR and AR in procedural training, it omitted the broader category of XR, which also includes MR. Additionally, the review focuses more on professional training and patient empowerment, with limited coverage of broader educational contexts, such as undergraduate medical education, theoretical learning and interprofessional collaboration. An expert commentary by Kok *et al* further reinforces the potential of immersive XR environments to address specific educational gaps in radiation oncology, offering practical implementation frameworks and design considerations to align XR modalities with pedagogical goals.^23^ These gaps leave an opportunity to explore XR’s full potential in transforming radiation oncology education, including its impact on curriculum development, knowledge dissemination and remote learning opportunities.

While patient-centred applications are important, the current review shifts the focus back to educational applications for medical trainees. Notably, XR applications in radiation oncology education were first explored in the context of training radiation therapists, making this professional group particularly relevant to the review.^23 24^ In addition, the field includes diverse learners such as medical students, radiation oncology residents, radiation oncologists, clinical oncologists in the UK, radiation therapists (RTTs, who specialize in the planning and delivery of therapeutic radiation to treat cancer patients), medical physicists (ROMPs, or Radiation Oncology Medical Physicists, who ensure the accurate and safe use of radiation technology through equipment calibration, treatment planning, and quality assurance), nurses and allied health professionals involved in cancer care.

By examining how XR technologies are used to enhance clinical decision-making, collaborative reasoning and team-based learning in radiation oncology education, this review will contribute to filling the gap identified in the literature on the broader applications of XR in medical education. This review will build on the methodological framework established by Arksey and O'Malley, with enhancements from Levac *et al*, and will follow the Preferred Reporting Items for Systematic Reviews and Meta-Analyses extension for Scoping Reviews (PRISMA-ScR) guidelines for reporting.^25^,^27^ The primary aim of this review is to assess the extent and type of evidence available on the use of XR in radiation oncology education, identifying key concepts, themes and knowledge gaps that could inform future research and practice.

## Methods

This scoping review will adhere to the PRISMA-ScR guidelines to ensure comprehensive and systematic reporting.^25^ The methodology will follow the Arksey and O’Malley framework, enhanced by the refinements proposed by Levac et al., and will incorporate a consultation phase to integrate expert perspectives.^26 27^ The iterative review process will encompass six key steps: (1) identifying research questions; (2) identifying relevant studies; (3) study selection; (4) data extraction; (5) collating, summarising and reporting results; and (6) consultation exercise. In this review, we will also evaluate methodological quality during the data extraction phase.

### Identifying research questions

The primary aim of this scoping review is to map the existing literature on the use of XR technologies—including VR, AR, and MR—in radiation oncology education. This review seeks to identify how these technologies are currently being used in educational settings, evaluate their effectiveness in enhancing learning outcomes and explore barriers to their adoption. The following research questions guide this review:

1. What are the types of XR technologies (including VR, AR and MR) currently being used in radiation oncology education?
2. How effective are these XR technologies in enhancing learning outcomes in both clinical and theoretical aspects of radiation oncology education?
3. What are the barriers and facilitators to the adoption of XR technologies in radiation oncology education, particularly in undergraduate medical education, professional training and interprofessional collaboration?
4. What are the current methodologies used to evaluate the impact of XR technologies in radiation oncology education, and what opportunities exist for standardising these assessments?
5. How can XR technologies be leveraged to support remote and flexible learning in radiation oncology education, particularly in response to limitations in physical clinical access?

### Identifying relevant studies

A comprehensive search strategy will be employed to identify relevant studies across multiple databases, including MEDLINE (via PubMed), SciVerse Scopus (Elsevier) and Web of Science. Grey literature, such as reports, theses and conference proceedings, will also be included to capture a broader range of evidence. The search strategy will use keywords and Medical Subject Headings terms related to extended reality, radiation oncology and education, such as “Extended Reality” OR “XR”; Virtual Reality” OR “VR”; Augmented Reality” OR “AR”; Mixed Reality” OR “MR”; Radiation Oncology”; and Medical Education” OR “Training” (online supplemental appendix I). Search results will be filtered for English-language studies published from January 2013 to the present. The year 2013 was selected as the start date due to the emergence of widely accessible XR devices, such as the Oculus Rift Developer Kit, which catalysed the development of XR tools in healthcare education.^28 29^ Reference lists of included sources will also be screened for additional relevant studies to capture any studies that might have been missed in the database searches.

### Study selection

All citations retrieved from the searches will be imported into EndNote 20 (Clarivate Analytics, Pennsylvania, USA) for management. The inclusion criteria for this review are (1) studies focusing on radiation oncology education, (2) scoping reviews, (3) articles written in English, (4) full-text, peer-reviewed journal articles and (5) publications from January 2013 to the present. Exclusion criteria will include protocols, abstracts, conference papers, symposiums, editorials, book chapters, brief reports, comments, grey literature and multidisciplinary studies with trivial or non-significant discussion of radiation oncology education.

The inclusion and exclusion criteria will be determined using the **PCC framework** (population, concept, context):

- **Population**: The target population for this scoping review includes individuals engaged in radiation oncology education and training. This encompasses undergraduate medical students, radiation oncology trainees and residents, and practising professionals such as radiation therapists, medical physicists and clinical oncologists (eg, as designated in the UK). In addition, the review includes members of multidisciplinary healthcare teams involved in the delivery of radiation therapy, including oncology nurses and allied health professionals. This definition reflects the interprofessional nature of modern oncology education and aims to capture all relevant learner groups exposed to XR-based educational interventions in radiation oncology contexts.
- **Concept**: XR technologies used in educational settings, including clinical simulations, theoretical training and collaborative learning.
- **Context**: Studies conducted in academic institutions, medical schools, hospitals and specialised training centres for radiation oncology.

Exclusion criteria will include studies focused exclusively on patient education, defined as educational interventions directed at patients rather than healthcare professionals, as well as non-educational use of XR (eg, treatment planning), and reviews lacking primary data.

Two reviewers will independently screen the titles and abstracts of all retrieved citations to identify potentially relevant studies. This will be followed by a full-text review of the selected studies to confirm their eligibility. A consensus on the selection criteria will be established in an initial meeting, with frequent discussions held to resolve discrepancies. In cases where disagreements persist, a third reviewer will be consulted. The screening process will be documented using a PRISMA flow diagram to provide a clear illustration of the study selection process.

### Data extraction

A standardised data abstraction form will be developed and pilot tested on a sample of studies to ensure comprehensive coverage and consensus among reviewers. The data extraction process will involve collecting detailed information on various aspects of each included study. This will include study characteristics (author, year, country); population (number of participants, education level); type of XR technology used (VR, AR, MR); educational setting (clinical simulation, theoretical instruction, etc); learning outcomes (knowledge acquisition, skill development, confidence levels); methodologies used to evaluate XR’s effectiveness; and barriers and facilitators to XR adoption.

The extracted data will be continuously reviewed and refined to ensure consistency. Two reviewers will independently extract data from each included study to ensure accuracy and consistency. Given the diverse themes in radiation oncology education research, an inductive thematic framework approach will be used for coding the data. Initially, the research team will analyse a subset of full-text publications to identify emerging themes. This will be followed by discussions to refine and finalise a coding framework. All included publications will then be coded according to this framework. Discrepancies in coding will be discussed and resolved by consensus, with a third reviewer involved if necessary. Extracted data will be organised and presented using Microsoft Excel (Microsoft, Redmond, USA).

### Evaluating methodological quality

The extracted data will be collated and analysed to map the types of XR technologies used in radiation oncology education, the contexts in which they are applied and the educational outcomes reported. Descriptive statistics will be used to present key findings, while qualitative thematic analysis will be employed to synthesise common themes related to barriers to adoption, methodological approaches and opportunities for remote learning. The results will be reported in accordance with PRISMA-ScR guidelines.

### Collating, summarising and reporting results

The collated data will be analysed and summarised to provide a comprehensive overview of the current research landscape in radiation oncology education. A descriptive numerical analysis will be used to summarise the coded data, providing quantitative insights into the volume and distribution of scoping reviews in radiation oncology education. Figures, tables and narrative content will be employed to present the findings clearly and effectively.

The results will highlight the themes, objectives and methodologies of existing scoping reviews in radiation oncology education. This synthesis will identify the strengths and weaknesses of current educational strategies, pinpointing gaps and areas needing further research. The findings will be categorised using the Arksey and O’Malley framework, with additional insights derived from the thematic analysis.

### Consultation exercise

To enhance the robustness and relevance of the review findings, we will conduct a consultation exercise with experts in radiation oncology education, medical education and XR technology. While experts affiliated with Chang Gung Memorial Hospitals (Medical Education Research Centre) and the University of Sydney (Faculty of Medicine and Health) will be among the invited participants due to existing academic collaborations, recruitment will not be limited to these institutions. Experts will be purposively identified through professional networks and institutional affiliations based on their demonstrated academic or clinical contributions to XR-enhanced education or radiation oncology training.

Eligibility criteria for participation include a minimum of 5 years of experience in relevant teaching, research or curriculum development roles. Structured interviews or focus group discussions will be conducted virtually using secure platforms such as Zoom (online supplemental appendix II). Each session will be facilitated by experienced moderators familiar with qualitative research methods and the content area. Informed consent will be obtained from all participants, and discussions will be audio-recorded (with consent) to ensure accuracy. Appropriate language support, including bilingual facilitation, will be provided as needed to accommodate international participants. Potential risks, particularly those related to data privacy and confidentiality, will be clearly communicated, and all data will be anonymised and securely stored.

### Ethics and dissemination

This protocol does not require ethics approval at the current stage as it involves only a planned scoping review of publicly available literature. Ethics approval is not required for the literature review component of this study. However, ethical approval will be obtained prior to the commencement of the consultation phase, in alignment with institutional research ethics guidelines. The consultation will involve experts from radiation oncology education, medical education and XR technology, ensuring their insights are incorporated ethically and responsibly. Consent will be documented through written consent forms or secure e-signature platform with documented confirmation, depending on the mode of consultation (eg, online or in-person) and in accordance with the ethics committee’s approved procedures. This ensures that participant contributions are voluntary, informed and appropriately safeguarded throughout the engagement process.

The dissemination plan includes publishing the results of this scoping review in high-impact, peer-reviewed journals relevant to both medical education and radiation oncology. Additionally, the findings will be presented at major conferences, including those focusing on medical education, technology-enhanced learning and oncology. To maximise the practical application of the findings, tailored executive summaries will be created for diverse stakeholder groups, including university educators, healthcare providers and policymakers. These summaries will also be shared in practice-oriented journals and distributed through professional networks to ensure that the insights reach those directly involved in curriculum development and decision-making processes.

### Patient and public involvement

Patients and the public were not involved in the design, conduct or dissemination plans of this scoping review.

## Discussion

This comprehensive and systematic approach to synthesising the existing literature on XR in radiation oncology education aims to provide valuable insights into current practices, identify critical research gaps and suggest methodological improvements. The outcomes of this review are expected to contribute significantly to advancing educational strategies, informing policy and ultimately improving the quality of both education and patient care in the field of radiation oncology.

Based on the anticipated scope of included studies, the review is expected to identify common themes across XR implementations, including (1) types of XR modalities used (VR, AR, MR); (2) target learner populations (eg, radiation oncology trainees, RTTs, medical students); (3) educational settings (clinical simulation vs didactic teaching); (4) learning objectives and outcome measures; and (5) implementation barriers and enablers (eg, technological infrastructure, faculty training, cost).

The review will also explore methodological approaches to evaluating XR, such as pre-knowledge/post-knowledge assessments, learner satisfaction, skill transfer metrics and qualitative feedback. Strengths of XR-based education are likely to include enhanced spatial understanding, immersive learning experiences and increased learner engagement, particularly in complex or high-stakes procedures. Conversely, limitations may include technological barriers, variability in instructional design and lack of long-term outcome data.

However, this study has several limitations. First, the exclusion of non-English language literature may introduce language bias and limit the generalisability of findings, especially from non-English-speaking countries with active XR adoption. Second, the review excludes grey literature such as industry reports and conference proceedings, which may contain relevant emerging applications not yet captured in peer-reviewed publications. Third, as a scoping review, the analysis will be descriptive and exploratory; it will not include formal quality appraisal of the included studies, which may limit the ability to assess the strength of the evidence. Fourth, while the planned consultation exercise will provide valuable expert insights, it is not intended to generate generalisable qualitative data and will be subject to the limitations of purposive sampling. Lastly, due to the rapid pace of technological change in XR, findings may become outdated quickly, necessitating periodic updates to maintain relevance.

By synthesising these dimensions, the review will offer structured guidance for educators and institutions considering the integration of XR into radiation oncology training. The findings will help clarify not only where XR is currently applied, but also where it may be most beneficial—such as remote learning, interprofessional education and procedural rehearsal. These insights are intended to inform the development of contextually appropriate and evidence-informed curricula.

## Supplementary material

10.1136/bmjopen-2024-094791 online supplemental file 1
