# Supplementary material for: Mapping the use of extended reality (XR) in radiation oncology education: a scoping review protocol
Source: BMJ Open. 2025 Jun 16;15(6):e094791. doi: 10.1136/bmjopen-2024-094791 (PMC12314805; doi:10.1136/bmjopen-2024-094791)
Supplement: online supplemental file 1 [file bmjopen-15-6-s001.docx]

### Appendix I: Search strategy

| **Database** 1 | MEDLINE (PubMed) |
| --- | --- |
| **Limits** | In: title, abstract, original title, name of substance word, subject heading word, keyword heading word, protocol supplementary concept word, rare disease supplementary concept word, unique identifier |
| **Search String** | ("Extended Reality" OR "Virtual Reality" OR "Augmented Reality" OR "Mixed Reality" OR XR OR VR OR AR OR MR).mp AND ("radiation oncolog*" OR "radiotherap*" OR “radiation therapy").mp AND (educat* or student* or train*).mp. |
|  |  |
| **Database** 2 | SciVerse Scopus (Elsevier) |
| **Limits** | In: Article Title, Abstract, Keywords  Published: "2013-2022" |
| **Search String** | ((TITLE-ABS-KEY ( “Extended Reality” ) OR TITLE-ABS-KEY (“Virtual Reality”) OR TITLE-ABS-KEY ( “Augmented Reality”) OR TITLE-ABS-KEY (“Mixed Reality”) OR TITLE-ABS-KEY ( XR ) OR TITLE-ABS-KEY (VR) OR TITLE-ABS-KEY (AR) OR TITLE-ABS-KEY (MR)) AND (TITLE-ABS-KEY ( radiotherp* ) OR TITLE-ABS-KEY ( radiation AND therapy ) OR TITLE-ABS-KEY ( radiation AND oncolog* ) AND TITLE-ABS-KEY (educat*) OR TITLE-ABS-KEY (student*) OR TITLE-ABS-KEY (train*))) |
|  |  |
| **Database** 3 | Web of Science |
| **Limits** | In: Article Title, Abstract, Keywords |
| **Search String** | (TS=“medical  ((TS= “Extended Reality” ) OR TS=“Virtual Reality”) OR TS= “Augmented Reality”) OR TS=“Mixed Reality”) OR TS= XR ) OR TS=VR) OR TS=AR) OR TS=MR)) AND (TS= radiotherp* ) OR TS= radiation AND therapy ) OR TS= radiation AND oncolog* ) AND TS=educat*) OR TS=student*) OR TS=train*))) |

### Appendix II. Topic Guide for Consultation Interviews

**Purpose:**
This topic guide will be used in semi-structured interviews conducted as part of the consultation phase of a scoping review. The objective is to gain insights from experts in radiation oncology, medical education, and XR technology to ensure that the review findings are interpreted in ways that are relevant, contextualized, and informed by practice.

**Interview Approach**

Following the guidance of Lichtman (2023), the interviews will be:

- Conversational but purposeful, allowing flexibility for participants to elaborate on areas of expertise;
- Open-ended in nature, encouraging reflection and depth;
- Organized thematically, aligned with the goals of the review.

Each interview will last approximately 30–45 minutes and be conducted in person or online (e.g., Zoom). Interviews will be recorded (with consent) and transcribed for analysis.

**Interview Topics and Sample Questions**

**I. Opening and Context Setting**

- Can you briefly tell me about your background and role in relation to radiation oncology or XR education?

**II. Current Practices and Perceptions**

- What types of XR tools (VR, AR, MR) have you seen used effectively in training or instruction?
- How do you perceive the value of XR compared to other educational methods?
- In your view, what are the educational challenges in radiation oncology that XR could help address?

**III. Implementation and Evaluation**

- What are the major barriers to implementing XR in your educational or clinical setting?
- What kinds of support (institutional, technical, pedagogical) are needed to integrate XR more effectively?
- How should we assess the educational impact of XR in radiation oncology? Are there any indicators or outcomes you consider important?

**IV. Broader Implications and Recommendations**

- Do you think XR has a role in promoting interprofessional or remote education in radiation oncology?
- What would you advise for educators or curriculum designers who are just beginning to adopt XR tools?
- Are there any risks, ethical concerns, or unintended consequences of XR in education that we should be aware of?

**V. Closing**

- Is there anything else you believe we should consider in our review?
