# Supplementary material for: Mapping the use of extended reality (XR) in radiation oncology education: a scoping review protocol
Source: BMJ Open. 2025 Jun 16;15(6):e094791. doi: 10.1136/bmjopen-2024-094791 (PMC12314805; doi:10.1136/bmjopen-2024-094791)
Supplement: Supplementary file 2 [file bmjopen-2024-094791.reviewer_comments.pdf]

PEER REVIEW HISTORY

BMJ Open publishes all reviews undertaken for accepted manuscripts. Reviewers are asked to complete a checklist review form and are provided with free text boxes to elaborate on their assessment. These free text comments are reproduced below.

ARTICLE DETAILS

Title (Provisional)

Mapping the Use of Extended Reality (XR) in Radiation Oncology Education: A Scoping Review Protocol

Authors

Lee, Ching-Hsin; Chen, Po-Jui; Lai, Hung-Yi; Chen, Mi-Mi; Yau, Sze-Yuen; Lee, Ching-Yi

VERSION 1 - REVIEW

**Reviewer**	**1**
**Name**	**Morris, Lucinda**
**Affiliation**	**University of New South Wales**
**Date**	**22-Jan-2025**
**COI**	**None**

I commend the authors on emanrking on this study assesing the current use of XR in Radiation Oncology Education. Overall this proposal is well written and the methodology is appropriate.

There are several comments/revisions that need adressing:

- Page 6 line 56 : "Specifically, the review will seek to answer the following questions" but no questions are listed after this, but seem to be later in line 33 page 7. In Methods it states databases searched are PubMed, Embase, MEDLINE, and Scopus. In Appendix only Pubmed, Elsevier and Web of Science are listed. This needs correction.

- Page 7, line 49 – exclusion of studies "focused exclusively on patient education, non-educational use of XR (e.g., treatment planning)" This needs further clarification. Does this mean education focussed on educating patients, or education directed specifically at patients? And if so why or why not was this chosen? If patients are excluded as learners, then why?

- Population is listed as "Medical students, radiation oncology trainees or professionals, and multidisciplinary healthcare teams". Setting a scope for the learner population is critical. Currently the target learner population needs to be much more clearly defined – which RO

professionals? RTTs, ROMPS, nurses? Allied health in cancer care? Other cancer specialties (e.g. medical oncology, haematology, surgery, radiology, pathology etc)? Other non-cancer specialties or healthcare professions? (primary care physicians) And justification for selecting each of these groups.

- Also if this is a global scoping review, will Clinical oncologists (e.g. in the UK) be included?

- Consultation Exercise will involved "Experts from Chang Gung Memorial Hospitals (Medical Education Research Centre) and the University of Sydney (Faculty of Medicine and Health)"

Further details are required:

• How will these experts be recruited? What is the consent/recruitment process/inclusion/exclusion criteria for participants?

• Why are only these two centres selected?

• What are the selection criteria as "experts" and what defines an expert in RO education?

• How will this group discussion data be collected / analysed? No methodology for this is outlined.

• What if any risks are there for participants

• How will the session take place, who will moderate, how might possible language barriers be navigated?

- Ethics. It is stated that "Ethics approval and consent to participate- The ethical approval or consent is not required in this study due to the nature of data extraction from published studies" However on page 9 line 26 it is stated that ethics approval with be sought. Please clarify / make consistent.

Lastly, review and reference to "Kok DL, Dushyanthen S, Peters G, Sapkaroski D, Barrett M, Sim J, Eriksen JG. Virtual reality and augmented reality in radiation oncology education–A review and expert commentary. Technical Innovations & Patient Support in Radiation Oncology. 2022 Dec 1;24:25-31." Is recommended.

Reviewer 2

Name Lastrucci, Andrea

Affiliation Department of Allied Health Professions, Azienda Ospedaliero-Universitaria Careggi

Date 01-Apr-2025

COI None

Your review protocol is well-structured, and the topic investigated is highly relevant. However, some improvements are needed to enhance its originality and make it truly innovative in this field.

In the Study Selection section under Population, I suggest extending the search to include healthcare professionals working in radiotherapy, such as radiation therapists. The first virtual reality software applications were initially developed for radiation therapy students, making their inclusion relevant. Given this proposed expansion, I also ask you to update the Introduction accordingly, including relevant references beyond medical students.

Additionally, please provide a rationale for selecting January 2013 as the cut-off date for the search.

The Discussion section needs improvement by detailing the expected outcomes of the review more comprehensively. Try to identify and elaborate on common themes that will likely be addressed in the review. Furthermore, I suggest conducting an analysis of the strengths and weaknesses of each key aspect you plan to cover. This will enhance the depth of the discussion and provide a more structured evaluation of the findings.

VERSION 1 - AUTHOR RESPONSE

Reviewer 1: Dr. Lucinda Morris
Comment 1: *Page 6 line 56: "Specifically, the review will seek to answer the following questions" but no questions are listed after this, but seem to be later in line 33 page 7.*

Response:
We thank the reviewer for this helpful observation. We have deleted the incomplete sentence from the Introduction. The full list of research questions is now presented only once, in the "Identifying Research Questions" subsection of the Methods, where they are appropriately contextualized and clearly stated (Page 6-7 line 172-192).

Comment 2: *In Methods it states databases searched are PubMed, Embase, MEDLINE, and Scopus. In Appendix only Pubmed, Elsevier and Web of Science are listed. This needs correction.*

Response:
Thank you for pointing out the inconsistency. We have revised the manuscript to ensure that database names are used accurately and consistently throughout. The final list of databases now reads: MEDLINE (via PubMed), SciVerse Scopus (Elsevier), and Web of Science, in alignment with their official naming conventions. References to Embase have been removed to match the actual search strategy.

Comment 3: *Page 7, line 49 – exclusion of studies "focused exclusively on patient education, non-educational use of XR (e.g., treatment planning)" This needs further clarification. Does this mean education focussed on educating patients, or education directed specifically at patients? And if so why or why not was this chosen? If patients are excluded as learners, then why?*

Response:
We have revised the exclusion criterion to clarify that this refers to studies in which XR is used solely for the purpose of educating patients, rather than training healthcare professionals (Page 8 line 236-239). The rationale for excluding these studies has been explicitly stated: the scope of this review is focused on professional education and training rather than patient-facing educational interventions.

Comment 4: *Population is listed as "Medical students, radiation oncology trainees or professionals, and multidisciplinary healthcare teams". Setting a scope for the learner population is critical. Currently the target learner population needs to be much more clearly defined – which RO professionals? RTTs, ROMPS, nurses? Allied health in cancer care? Other cancer specialties (e.g. medical oncology, haematology, surgery, radiology, pathology etc)? Other non-cancer specialties or healthcare professions? (primary care physicians) And justification for selecting each of these groups.*

Response:
We thank the reviewer for this important suggestion. In response, we have revised the Population subsection within the PCC framework to provide a more specific, sentence-based definition. The revised text now states:

"*The target population for this scoping review includes individuals engaged in radiation oncology education and training. This encompasses undergraduate medical students, radiation oncology trainees and residents, and practicing professionals such as radiation therapists, medical physicists, and clinical oncologists (e.g., as designated in the UK). In addition, the review includes members of multidisciplinary healthcare teams involved in the delivery of radiation therapy, including oncology nurses and allied health professionals. This definition reflects the interprofessional nature of modern oncology education and aims to capture all relevant learner groups exposed to XR-based educational interventions in radiation oncology contexts.*" (Page 8 line 221-231).

Comment 5: *Also if this is a global scoping review, will Clinical oncologists (e.g. in the UK) be included?*

Response:
Yes, clinical oncologists practicing in the UK have been explicitly included as part of

the target learner population. This clarification has been added to both the Introduction and the PCC definition in the Methods section.

Comment 6: *Consultation Exercise will involved "Experts from Chang Gung Memorial Hospitals (Medical Education Research Centre) and the University of Sydney (Faculty of Medicine and Health)"*

Further details are required:

- *How will these experts be recruited? What is the consent/recruitment process/inclusion/exclusion criteria for participants?*
- *Why are only these two centres selected?*
- *What are the selection criteria as "experts" and what defines an expert in RO education?*
- *How will this group discussion data be collected / analysed? No methodology for this is outlined.*
- *What if any risks are there for participants*
- *How will the session take place, who will moderate, how might possible language barriers be navigated?.*

Response:

We thank the reviewer for this detailed and thoughtful comment. In response, we have substantially revised the Consultation Exercise section in the Methods to clarify the recruitment process, participant eligibility, data collection procedures, and risk mitigation strategies (Page 10 line 292-312).

We now state that expert participants will be purposively identified through professional networks and institutional affiliations based on their demonstrated academic or clinical contributions to XR-enhanced education or radiation oncology training. While experts from Chang Gung Memorial Hospitals and the University of Sydney will be included due to ongoing academic collaborations, recruitment will not be limited to these two institutions. This broader approach ensures greater diversity of perspectives and enhances the generalizability of insights gained through the consultation.

Eligibility for participation requires a minimum of five years of relevant experience in teaching, research, or curriculum development within radiation oncology education or XR-enhanced instruction. Informed consent will be obtained from all participants prior to engagement in the study. No exclusion criteria will be applied beyond the relevance of expertise to the review topic.

Structured interviews or focus group discussions will be conducted virtually using secure online platforms such as Zoom. Sessions will be facilitated by experienced moderators with expertise in qualitative research and the relevant content domains. With participant consent, discussions will be audio-recorded to ensure accuracy and

later transcribed for analysis. Data will be analyzed thematically to identify expert insights, validate the findings of the scoping review, and explore implications for future educational practices.

Risks to participants are expected to be minimal and primarily related to data privacy and confidentiality. These risks will be clearly communicated during the consent process, and all data will be anonymized and securely stored. Language barriers will be mitigated by offering bilingual facilitation or translation support where necessary, to ensure inclusivity and clarity for international participants.

We believe these enhancements strengthen the methodological transparency and ethical rigor of the consultation phase, in alignment with scoping review best practices.

Comment 7: *Ethics. It is stated that "Ethics approval and consent to participate- The ethical approval or consent is not required in this study due to the nature of data extraction from published studies" However on page 9 line 26 it is stated that ethics approval with be sought. Please clarify / make consistent.*

Response:
We have clarified the distinction between the two components of the study (Page 10 line 314-318). Ethics approval is not required for the scoping review of published literature. However, ethics approval will be sought for the consultation phase involving expert participants. This distinction has been explicitly stated in the Ethics and Dissemination section.

Comment 8: *Lastly, review and reference to "Kok DL, Dushyanthen S, Peters G, Sapkaroski D, Barrett M, Sim J, Eriksen JG. Virtual reality and augmented reality in radiation oncology education–A review and expert commentary. Technical Innovations & Patient Support in Radiation Oncology. 2022 Dec 1;24:25-31." Is recommended.*

Response:
We thank the reviewer for this recommendation. We have now incorporated Kok et al. (2022) into the Introduction to strengthen our contextual framing of XR applications in radiation oncology education. The reference has also been added to the reference list (Page 10 line 135-139 & line 144-147)

Reviewer 2: Dr. Andrea Lastrucci
Comment 1: *In the Study Selection section under Population, I suggest extending the search to include healthcare professionals working in radiotherapy, such as radiation therapists. The first virtual reality software applications were initially developed for radiation therapy students, making their inclusion relevant.*

Response:
Thank you for this important recommendation. In response, we have revised the Population subsection of the PCC framework to explicitly include radiation therapists (RTTs) and other healthcare professionals working in radiotherapy. The revised population definition now reads:

"The target population for this scoping review includes individuals engaged in radiation oncology education and training. This encompasses undergraduate medical students, radiation oncology trainees and residents, and practicing professionals such as radiation therapists, medical physicists, and clinical oncologists (e.g., as designated in the UK). In addition, the review includes members of multidisciplinary healthcare teams involved in the delivery of radiation therapy, including oncology nurses and allied health professionals." (Page 8 line 221-231)

Comment 2: *Given this proposed expansion, I also ask you to update the Introduction accordingly, including relevant references beyond medical students.*

Response:
We appreciate this suggestion and have revised the Introduction to reflect the broader application of XR technologies in radiation oncology education beyond undergraduate learners. Specifically, we now acknowledge that some of the earliest XR applications in healthcare education were developed for training radiation therapists. To support this statement, we have incorporated relevant references, including studies by Sapkaroski et al. (2020), Kok et al. (2022), and Bridge et al. (2014), which document the historical development and application of virtual reality in radiotherapy education.

1 Bridge, P., Gunn, T., Kastanis, L., & Pack, D. (2014). The use of simulation in radiotherapy education: A review of the literature. *Journal of Medical Radiation Sciences, 61*(3), 259–263. https://doi.org/10.1002/jmrs.75
2 Kok, D. L., Dushyanthen, S., Peters, G., Sapkaroski, D., Barrett, M., Sim, J., & Eriksen, J. G. (2022). Virtual reality and augmented reality in radiation oncology education–A review and expert commentary. *Technical Innovations & Patient Support in Radiation Oncology, 24*, 25–31. https://doi.org/10.1016/j.tipsro.2022.05.001
3 Sapkaroski, D., Mundy, M., & Dimmock, M. R. (2020). Virtual reality versus conventional clinical role-play for radiographic positioning training: A students' perception study. *Radiography, 26*(1), 57–62. https://doi.org/10.1016/j.radi.2019.08.001

Comment 3: *Please provide a rationale for selecting January 2013 as the cut-off date for the search.*

Response:
Thank you for this important point. We have now included a rationale in the Methods section under “Study Selection” to justify the 2013 cut-off. The revised text explains

that January 2013 was chosen as the start date to coincide with the emergence of widely accessible XR devices such as the Oculus Rift Developer Kit, which catalyzed a marked increase in XR research and application in healthcare education. This justification is supported by literature (e.g., Freina & Ott, 2015; Radianti et al., 2020), which documents the technological and scholarly inflection point beginning in that period.

1 Freina, L., & Ott, M. (2015). A literature review on immersive virtual reality in education: State of the art and perspectives. In *The International Scientific Conference eLearning and Software for Education* (Vol. 1, pp. 133–141).
2 Radianti, J., Majchrzak, T. A., Fromm, J., & Wohlgenannt, I. (2020). A systematic review of immersive virtual reality applications for higher education: Design elements, lessons learned, and research agenda. *Computers & Education, 147*, 103778. https://doi.org/10.1016/j.compedu.2019.103778

Comment 4: *The Discussion section needs improvement by detailing the expected outcomes of the review more comprehensively. Try to identify and elaborate on common themes that will likely be addressed in the review.*

Response:
We appreciate the reviewer's suggestion and have revised the Discussion section to more fully articulate the anticipated outcomes of the review (Page 11-12). The updated text identifies and elaborates on likely thematic areas such as XR modality types (VR, AR, MR), target learner populations, educational settings (e.g., clinical simulation vs. didactic instruction), learning outcomes, and common barriers and enablers of implementation. This revision provides a clearer preview of the dimensions that will be mapped in the review and strengthens the alignment between the review objectives and its anticipated contributions.

Comment 5: *I suggest conducting an analysis of the strengths and weaknesses of each key aspect you plan to cover. This will enhance the depth of the discussion and provide a more structured evaluation of the findings.*

Response:
Thank you for this valuable recommendation. In response, we have expanded the Discussion section to outline a preliminary framework for analyzing strengths and limitations across key domains of the included studies—specifically regarding XR technology type, instructional design quality, learner population alignment, and implementation feasibility. This approach will enable a more structured synthesis and highlight both promising practices and methodological gaps in the field.
